# Applying Econometrics to the Carbon Dioxide “Control Knob”

**DOI:** 10.1100/2012/761473

**Published:** 2012-05-03

**Authors:** Timothy Curtin

**Affiliations:** Emeritus Faculty, Australian National University, Canberra, ACT 0200, Australia

## Abstract

This paper tests various propositions underlying claims that observed global temperature change is mostly attributable to anthropogenic noncondensing greenhouse gases, and that although water vapour is recognized to be a dominant contributor to the overall greenhouse gas (GHG) effect, that effect is merely a “feedback” from rising temperatures initially resulting *only* from “non-condensing” GHGs and not at all from variations in preexisting naturally caused atmospheric water vapour (i.e., [H_2_O]). However, this paper shows that “*initial radiative forcing*” is not exclusively attributable to forcings from noncondensing GHG, both because atmospheric water vapour existed before there were any significant increases in GHG concentrations or temperatures and also because there is no evidence that such increases have produced measurably higher [H_2_O]. The paper distinguishes between forcing and feedback impacts of water vapour and contends that it is the *primary* forcing agent, at much more than 50% of the total GHG gas effect. That means that controlling atmospheric carbon dioxide is unlikely to be an effective “control knob” as claimed by Lacis et al. (2010).

## 1. Introduction: Previous Econometric Modelling

The main technique used in this paper is econometric least squares regression analysis, which enables computation of the relative strength of proposed alternative and independent causal factors in determination of the dependent variable, temperature change. This procedure is not used in Solomon et al. [1] or by Schmidt et al. [2] and Lacis et al. [3]. Instead, they all rely on computer models of the climate system in which parameterized expressions for the main variables under consideration are first used to generate a simulation of the global climate, and when the average of an ensemble of such models generates some conformity with observations, the expressions for one or other of the noncondensing and condensing GHGs are removed in turn from their composite model, and thereby they estimate the relative strength of individual GHGs. However, the claims that only the noncondensing GHGs are the “forcing” agents, and that condensable water vapour has just a feedback role, are built into the models' alternate simulations, and do not constitute confirmatory evidence validating their hypothesis that the only role of water vapour and clouds is to “amplify the initial [sic] warming provided by the noncondensing GHGs, and in the process, account for the bulk of the total terrestrial greenhouse effect” [3–9]. For that, in the absence of controlled physical experiments like those of Tyndall [10], which are not possible at the global or regional levels with or without computer models, econometrics is essential.

Dessler and Davis [11, page 1] state that the water vapour feedback “is the process whereby an initial warming of the planet, caused, for example, by an increase in long-lived greenhouse gases, leads to an increase in the humidity of the atmosphere. Because water vapour is itself a greenhouse gas, this increase in humidity causes additional warming. This is the most powerful feedback in the climate system, with the capacity by itself to double [sic] the warming from carbon dioxide alone.” That claimed positive feedback is what explains how the IPCC's predicted global temperature increase for a doubling in [CO_2_] from the c.280 ppm in 1900 of 3°C (central value) to 560 ppm implies an increase of 2.3°C from the extra 60 percent in [CO_2_] from 2010, despite the observed only 0.83°C associated with the nearly 40 percent increase in [CO_2_] between 1900 and 2010 (Gistemp). This paper's regression analysis tests for the relative importance of changes in [CO_2_] and [H_2_O] and also as to which comes first, the former according to Dessler and Davis [11], or the latter, in “forcing” temperature changes.

Not many researchers have used time domain econometrics methods to analyze climate change. Stern and Kaufmann [12, page 412], Tol and de Vos [13], and Tol [14], are amongst the few that explicitly use econometric multi-variate regression analysis of time series data to investigate the causes of climate change…^1^

None of these papers addresses the respective proportions of condensing and noncondensing GHGs to the overall greenhouse effect, and none mention [H_2_O] as an independent variable with potential explanatory value for changes in temperature. Kaufmann et al. [15, 16] have made further use of econometric methods, and comment how “statistical models of the relationship between surface temperature and radiative forcing that are estimated from the observational temperature record often are viewed skeptically by climate modelers. One reason is uncertainty about what statistical models measure. Because statistical models do not represent physical linkages directly, it is difficult to assess the time scale associated with statistical estimates for the effect of a doubling in CO_2_ on surface temperature.” These papers' database regressions (Section 4) use a wide range of “physical linkages,” and the derived coefficients provide an ample resource for “assessing the time scale… for the effect of a doubling in CO_2_,” which could be more than a hundred years if their analysis is correct.^2^

Hegerl et al. [17], in AR4, [1] claimed that they would attempt to differentiate between climate changes “that result from anthropogenic and natural external forcings” (p.667). However, they do not report any regression results estimating the relative values of those forcings. They concede (p.668) that attribution studies seek to “assess whether the response to a key forcing, such as greenhouse gas increases, is distinguishable from that due to other forcings (Appendix 9A) and add that “these questions are typically investigated using a multiple regression of observations onto several fingerprints [sic] representing climate responses to different forcings… see Section 9.2.2.” However, there is no trace of the results of any such analysis anywhere in Hegerl et al. 2007, least of all in either their referenced Section 9.2.2 or their Appendix 9A. The latter (pp.744-745) does have a textbook account of multivariate regression but reports no results. Thus Hegerl et al. [12, p.666] provide no evidence for their assertion “greenhouse gas forcing has very likely caused *most* of the global warming over the last 50 years” where “very likely*”* means “more than 90 percent probability” [1, page 121], and “most” must mean at least more than 50 percent when only two independent variables are considered.^3^ Had these authors done some regression analysis, they could have been more precise, but they never did, nor do they report any by others.

Instead, for both Hegerl and Allen [18] and the many co-authors of the 11 papers cited by Hegerl et al. [17] of which Hegerl was the lead author, “attribution” consists of model outputs with imposed parameters of radiative forcing arising from [CO_2_] and other greenhouse gases.^4^ In practice, none of these papers perform any regression analysis of both natural and nonnatural forcings and ignore primarily “natural external forcings” like that from [H_2_O]. Hegerl and Allen [18] deal only with greenhouses gases and sulphur dioxide, and the latter is even more of anthropogenic origin (mainly comprising emissions from combustion of hydrocarbon fuels) than the former. It is true that sulphate aerosols are usually assumed to have a cooling effect, see Charlson and Wigley [19], but most sulphate aerosols (hereafter [SO_2_]) are of the same anthropogenic origin in time and place as emissions of CO_2_ although from time to major volcanic eruptions increase both [CO_2_] and [SO_2_], with only local effects in the case of the latter. The other papers cited by Hegerl et al. [17] adopt much the same approach. For example Hegerl et al. [20, page 632] consider only [CO_2_] and [SO_2_] with just this mention of solar irradiation at the top of the atmosphere (TOA): “We used only a greenhouse gas and a greenhouse gas-plus-aerosol signal pattern, since the solar response pattern could not be sufficiently separated from noise and the greenhouse gas pattern,” a curious conclusion in the light of the title of that paper.

Stott et al. [21, page 2]^5^ use what they call “optimal detection technology” to conclude that “increases in temperature observed in the latter half of the century have been caused by increases in anthropogenic greenhouse gases offset by cooling from tropospheric sulphate aerosols rather than natural variability…” They claim that their “technology” is simply “just least squares regression in which we estimate the amplitude in observed data of prespecified [i.e., modelled] patterns of climate change in space and time” [21, page 1], yet at no point does their paper report adjusted *R* ^2^ or any other standard regression statistics (e.g., sum of squares,* F*, coefficients, standard errors, *t*-statistics, or *P-value*s arising from their regressions). Nor does their paper report any of the standard tests (Durbin-Watson, Dickey-Fuller) for serial autocorrelation and thereby for spurious correlations, and least of all, any of the normal tests for multicollinearity. Moreover, these authors' “control simulation, in which *external* climate forcings…are kept constant to simulate [sic] natural *internal* variability, has been run for over 1700 years [sic] (our emphasis)” is contradictory.^6^

This paper uses overlooked NOAA-ESRL site-specific databases of statistics on a wider range of both human and natural climatic variables than is analyzed in any of the “detection and attribution” papers noted above. We show that a comprehensive analysis results in relegating [CO_2_] to insignificance as a determinant of climate change, and that atmospheric water vapour arising almost exclusively from nonhuman sources is by far the largest source of radiative forcing and temperature change. We thereby hope to achieve a better response to the Kaufmann et al. [15, 16] challenge noted above, however incompletely.  Section 2 provides an assessment of the appropriate specifications to be adopted for multivariate regression analysis of various models' climatic variables, while Section 3 outlines the paper's data sources. Section 4 reports its regression results, and the concluding Section 5 provides discussion of the implications of these results.

## 2. Methodology

Unlike mainstream climate science, which relies wholly on “general circulation models” (GCM) [4, page 749], few of which successfully hindcast the observational record without retrospective fine tuning of parameters, we seek to evaluate the following climate change models using only the observational record. That is represented by measures of monthly or annual temperatures (*T*), such as minimum, maximum, and mean, at various locations between 1960 and 2006, as potentially mostly determined by one of the following, including rising atmospheric concentration of greenhouse gases in general, represented by *x*_1_, [CO_2_] (following [13, page 96]), by variations in *x*_2_, solar surface radiation (SSR, in Watt hours per square meter, Wh/m^2^), and *x*_3_, atmospheric water vapour ([H_2_O], in cm.)

(1) $$\begin{array}{c} T=a+bx_{1}+cx_{2}+dx_{3}+ex\ldots_{n}+u\left[x\right]. \end{array}$$

Variable *u*[*x*] is an error or “noise” term that represents any failure of the linear combination of *x*_1_, *x*_2_, and *x*_3_ to account fully for *T*. However, because of substantial evidence of spurious correlations when regressing *T* on the independent variables in (1), we assess the similar hypothesis, that year on year *changes* in temperature are determined by year on year *changes *in those independent variables (see (4) below).

It is important to establish that the RHS variables in (1) are indeed independent of each other, so I run regressions of each of *x*_1,…*n*_ on each other in turn; for example, if *x*_1_ represents atmospheric water vapour [H_2_O] and *x*_2_ is [CO_2_], then we need to know if *x*_1_ is a function of *T* and *x*_2_:

(2) $$\begin{array}{c} x_{1}=a+fT+cx_{2}\ldots .ex_{\ldots n}. \end{array}$$

Clearly, total independence of the *T* and [CO_2_] variables is questionable, as on colder/hotter days offices and households are likely to use more heating/cooling, and if that involves burning of more hydrocarbon fuels, then large changes in *T* from ambient levels will affect additions to [CO_2_]. I have done tests (not reported here) which show that changes in [CO_2_] appear to have no impact on changes in [H_2_O]. The outcomes of the regression analysis of (1) and (2) are discussed below. Additional regression results may be found in the Supplementary Material available on line at doi: 10.1100/3012/761473. Although there has been general agreement that the *T* and [H_2_O] variables are independent, as “most of (the water vapour in the atmosphere) originates through evaporation from the ocean surface and is not influenced directly by human activity” ([22, page 23] see also IPCC, TAR, [23]), the view in IPCC AR4 [1] is that atmospheric water vapour is increasing because of the rises in temperature attributed to increasing [CO_2_] (see below for assessment of that claim).

There has been considerable debate since Granger and Newbold [24] on how best to ensure that OLS regression of the variables in (1) does not produce spurious correlations between the temperature and the independent variables *x*_1_, *x*_2_, and *x*_3_. Various tests have been devised to determine whether the variables are “stationary” or have “unit roots.” The presence of a unit root in a time series is considered to invalidate standard regression analyses because that series is no longer stationary, this being a necessary condition to ensure avoidance of spurious correlation^7^. For example, many time series in economics have a steady upward trend similar to that of the concentration of carbon dioxide in the atmosphere [CO_2_]—numbers of television sets, mobile phones, computers, and their broadband connections all show steady upward trends worldwide, but none of these trends can plausibly imply either direct or inverse causal relationships with [CO_2_] despite no doubt striking correlation coefficients between them and rising [CO_2_].

One widely applied solution to the problem of nonstationarity in time series is first to difference the series in question, by subtracting the present value of a variable from the previous value, and so on for all values in the series.^8^ A simple regression model is merely a *straight line *fitted to a scatter-plot of one variable versus another. So when there are debates as in Kaufmann and Stern [25] and Kaufmann et al. [15, 16] as to whether various statistics, such as local or global temperatures and other climate variables, have a unit root and thereby require cointegration, or are trend stationary, this means only that there is a problem in *system identification*. That means we have to determine whether we are looking at the output of a first order low pass filter (what the statisticians call *I*(0) or at the output of an integrator—as in *I*(1)). In the former, the variance is a constant (although the distribution may be around a linear trend), in the latter the variance is itself expanding (or inflating).

In this paper's *in situ* (local) model and its data these considerations are irrelevant. All that matters is that the data on changes in [CO_2_] and [H_2_O] and any other causative variables should be linearly independent. A key requirement—spelt out in rule (5) in the list below—is that this noise must have a constant variance over the distribution of samples; it must be *I*(0). We need only to take first differences if we have some reason to suppose that this noise is *I*(1), and because we do find evidence of multicollinearity when regressing the absolute values of the independent and dependent variables of interest, we focus here mostly on the results of regressions taking the first differences of both the dependent and the independent variables. However, “stationarizing” in this manner is not necessarily one of the general rules for successful application of regression analysis (and calculation of meaningful statistics subsequently).

In general, the various rules or conditions that must be satisfied for a valid regression are the following:

1. the predictor samples *x*_*t*1,2…*n*_  and *y*_*t*_ must be representative of the population that they are sampling;
2. the unknown *u*_*t*_ must have zero mean;
3. the predictors must be linearly independent;
4. the unknown *u*_*t*_ must be uncorrelated;
5. the unknown *u*_*t*_ must be samples from a random variable population with constant variance, or *homoscedastic. *

Evidently, there is no particular requirement that the vectors *x* and *y* of the respective data should conform to a time series with specific statistical properties. The noise variables *u*_*t*_ in (1) appear to be I(0), with uncorrelated zero mean and with no expansion of the variance, at least there is no evidence that they are not.

The aim is to establish if the level of [CO_2_] is or is not—the main explanatory variable of average global or local temperature—in some quasimonotonic relation. For simplicity we stick to basic linear regression.

The Mauna Loa Slope Observatory in Hawaii has provided a test range of CO_2_ from 315.71 ppm in April 1958 to 393.39 ppm in April 2011 and such current levels are confirmed by other measurements that started some years later, like those at Pt. Barrow in Alaska and elsewhere, including Cape Grim in Tasmania^9^. We may call this the independent “*x*_1_” variable. Let *y* represent the averaged annual temperature at either the global or some specific location. Is there a dependence *y* ≫ *f*(*x*)—or linearized about some operating point, does *y* ≫ *a* + *bx*?^10^ Perhaps so, but it makes no difference whatsoever in the testing whether *x* itself should exhibit a consistent rising trend (what engineers call a “ramp”) or whether it is noise-like. The condition to satisfy rule (1), that *x* should cover with reasonable uniformity the given range, is clearly satisfied. Where this paper seeks to make a useful advance is in proposing a multiple regression to include all the potential causes of “weather.”

One obvious candidate for determining mean maximum (i.e., day) temperature in addition to [CO_2_] has to be localized solar surface radiation SSR in Watt hours/sq. meter which I call here *x*_2_. If the sun shines on any given day of successive years more or less “vertically” (or with less albedo) at any one place, subject to the level of atmospheric water vapour at the same place, then the temperature is likely to vary with the respective variations in solar radiation at that place. Similarly, the level of [H_2_O] at any given time and place, closely related to the relative humidity (RH) that is well known to make any given temperature level seem “hotter” than otherwise, has a no more evident relationship with [CO_2_] than the level of solar surface radiation. That is because [CO_2_] is invariant across the globe, at all given times and places, while [H_2_O] varies enormously at any given latitudes and times.

Again for simplicity let us introduce this one further possible explanation as *z* ≫ *f*(*x*_1_, *x*_2_) or linearized *z* ≫ *a* + *bx*_1_ + *cx*_2_. Rule (3) says that formally there should be no linear dependence of *x*_1_ and *x*_2_, as that could produce multicollinearity and spurious correlations with temperatures *y*. There seems little risk of that with these variables. There is no reason why atmospheric water vapour and total watt hours of sun at any one location during any year would be coupled and connected to the level of [CO_2_] at that location in that year. Whether time series *x*_1_, *x*_2_ … .*x*_*n*_ and time series *y* exhibit nonstationarity or not is irrelevant and incidental when they are independent of each other, but, to be on the safe side, we provide standard tests for the presence or not of multicollinearity and show that there is no such presence in any of the regressions of our first differenced data.

What a first differencing exercise may usefully show is a better exhibition of a rising trend in temperature since the “noise” in the measurements hopefully has been reduced by introducing the additional independent variables using *x*_2_ … *x*_*n*_. Thus our multiple regression analysis seeks to remove or at least mitigate the scatter in annual temperature by testing if and when that scatter is linked to changes in solar surface radiation and other climatic variables such as [H_2_O], in the hope of revealing a better measurement of a linear trend in temperature (which would be otherwise nondiscernible for the data assembly in our selected sites).

Again, what really matters is the statistical property of the error sequence *u*_*t*_. We assume that this is an *I*(1) sequence, because there is evidence for autocorrelation and multicollinearity of the absolute data, and that is why we rely on first differenced data. In general we find that [CO_2_] plays at best a marginal role—and one that is usually statistically insignificant—in explaining the temperature changes at various locations in USA over the 47 years inclusive between 1960 and 2006 (when the NOAA discontinued reporting the data sets used here, although a similar but less comprehensive series with data from 1948 to 2011 for locations defined by their latitude and longitude is available from ESRL-NASA).^11^

## 3. Data Sources

The “BEST” data sets [26] are the latest attempts to “homogenize” the most widely used global temperature sets, namely, Gistemp, HadleyCRU, and NCDC, but exclude the ESRL-NOAA data that have attempted the same task since 1996 [27]. In Section 4 below I use the BEST data set for global assessment since 1990, and in Supplementary Material, the NASA-GISS Gistemp series since 1958. For *in situ* (local) analysis I use the ESRL-NOAA database which covers some 1,200 locations across the whole of the USA since 1990, and of these more than 200 have data extending back to 1960. However I report detailed results of such analysis for just Point Barrow in Arctic Alaska and Hilo in equatorial Hawaii (at the foot of Hawaii's Mauna Loa) and at Mauna Loa itself, where Keeling set up his [CO_2_] observatory in 1958. In Supplementary Material I also report regressions of data from various other locations.

Table 7 presents a specimen of the NOAA-ESRL raw data from Point Barrow, in the arctic circle at the northernmost tip of Alaska, where if [CO_2_] is to be significant anywhere, it has to be there, given mean temperatures that have always been negative since 1960, despite [CO_2_] levels there that are almost identical to those at Mauna Loa in Hawaii and elsewhere on the globe.^12^ Not only that, Barrow being in the Arctic Circle is a pristine site, far removed from confusing elements such as the urban heat island (UHI) effect, which is why it was selected as one of the gold standard locations for measurement of [CO_2_]. That is also why Keeling selected Mauna Loa for his first [CO_2_] measurement station, as it too is far away from other anthropogenic influences, at an altitude of 3,500 meters above sea level. 

## 4. Regression Results

### 4.1. Is Most of Observed Temperature Change due to Anthropogenic GHGs?

I first regress the global mean temperature (GMT) anomalies against the global annual values of the main climate variable evaluated by the IPCC Hegerl et al. [17] and Forster et al. [28] based on Myhre et al. [29], namely, the total radiative forcing of all the noncondensing greenhouse gases [RF]

(3) $$\begin{array}{c} \text{Annual}\;\left(\text{Tmean}\right)=a+b\left[\text{RF}\right]+u\left(x\right)\ldots . \end{array}$$

The results appear to confirm the findings of Hegerl et al. [17] with a fairly high *R* ^2^ and an excellent *t*-statistic (>2.0) and *P-*value (<0.01) but do not pass the Durbin-Watson test (>2.0) for spurious correlation (i.e., serial autocorrelation), see Table 1. This result validates the null hypothesis of *no statistically significant* influence of radiative forcing by noncondensing GHGs on global mean temperatures.^13^

**Figure 1:**
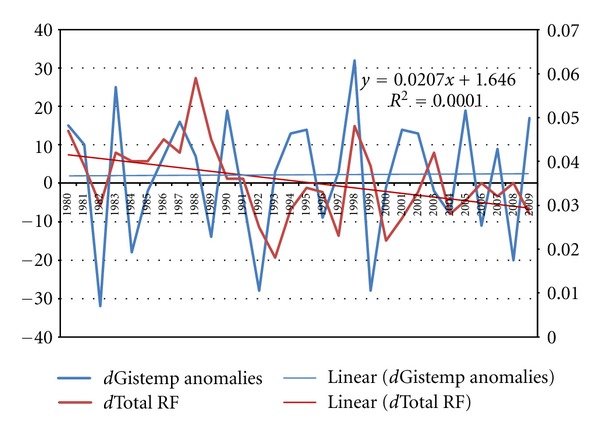
Plot of first differences in temperature anomalies and total radiative forcing (by all noncondensing GHGs). Source: Muller et al. [26].

Modifying (3) to represent first differences in both the dependent and independent variable,

(4) $$\begin{array}{c} \Delta \text{Annual}\;\left(\text{Tmean}\right)=a+b\left(\Delta \left[\text{RF}\right]\right)+u\left(x\right)\ldots . \end{array}$$

regression of year-on-year *changes* in GMT against those in [RF] passes the Durbin-Watson test statistic, but the adjusted *R* ^2^ statistic is now far below 0.5, so does not confirm the Hegerl et al. assertion (in Solomon et al. [1]) that “most” (at least more than 50 percent) of changes in GMT result from changes in [GHG] attributable to human causation (see Table 2 and Figure 1). The failure of the regression to reveal *any* contribution of changes in [GHG] to changes in Gistemp's GMT anomalies is obvious both from Figure 1 and from Table 2, which shows *total statistical insignificance *because with *t* < 2.0, and *P* > 0.05, the critical values are not attained. These results validate the null hypothesis from Hegerl et al. [17] that there is no discernible and statistically significant causation of global temperature change attributable to the radiative forcing from anthropogenic changes in noncondensing GHGs. 

The minimal level of *R* ^2^ indicates serious omitted variable bias, and this could be addressed by using the ESRL-NOAA data for precipitable water [H_2_O], with results shown in Table 3. Unfortunately, unlike the NOAA data sets for hundreds of locations in USA from 1960 to 2006, the ESRL-NOAA global reanalysis data sets exclude critical variables like solar surface radiation, and that explains why the minimal *R* ^2^ in Table 3 again indicates the absence of such variables and plausibly explains why the radiative forcing from [CO_2_] and [H_2_O] has such minimal statistical significance and can in no sense be described as “control knobs.”

Next, I use first differences regressions to include NOAA data on nonanthropogenic variables at various locations of atmospheric water vapor [H_2_O], and solar surface radiation in addition to [CO_2_] as the main atmospheric GHG^14^

(5) $$\begin{array}{cc} & \text{Annual}\left(T\max_{t1-t0},T\min_{t1-t0},{\text{AvD}T}_{t1-t0}\right)\\ & \;\;=a+b\left({\left[{\text{CO}}_{2}\right]}_{t1-t0}\right)+c\left({\text{AVGLO}}_{t1-t0}\right)\\ & \;\;\;+d\left({\text{H}}_{2}{\text{O}}_{t1-t0}\right)\ldots . \end{array}$$

I first run this model for mean *minimum* temperatures at Point Barrow in Alaska from 1960 to 2006 with results reported in Table 4. This model passes the autocorrelation (D-W > 2.0) and collinearity tests. The nonanthropogenic [H_2_O] variable is highly statistically significant in regard to what are night temperatures, at the better than 99 percent level, while the coefficient on the differenced [CO_2_] variable is barely positive and remains statistically insignificant. 

The adjusted *R* ^2^ in Table 4 is somewhat lower at 0.41 than the 0.63 in Table 2, so clearly there is still at least one omitted explanatory variable. As there is virtually no sunshine at Barrow for most of the winter, solar surface radiation is not a serious candidate, but obvious candidates include temperature variation arising from the ocean currents offshore of Pt Barrow, Arctic Ocean heat content, and decadal wind variability (see [30–33]). These variables are beyond the scope of this paper. However, if we now consider mean maximum temperatures at Barrow, then net solar surface radiation (“AVGLO”) and opacity of the sky (“OPQ”) (using their absolute values) can be added to the regression, with results shown in Supplementary material. There the unadjusted *R* ^2^ is 0.39, and only the [H_2_O] variable is statistically significant, accounting for more than 90 per cent of the changes in mean maximum temperature over the period 1960–2006, thereby going beyond the assertion by Schmidt et al. 2010 cited above that atmospheric water vapor accounts for only 50 per cent of the total greenhouse effect [2].

The conclusion from the limited model used in Tables 4 and 5 is that there can be a high degree of confidence that the increasing radiative forcing due to rising [CO_2_] at Pt Barrow attributable to anthropogenic emissions plays no role in explaining the climate there since 1960. We are also able to show that which proves to be the case for all the other locations where the same regression analysis is possible (see examples in the Supplementary Material). 

I now provide here one further site-specific regression analysis, for the Slope Laboratory at Mauna Loa itself (Table 6), close to the Equator while Barrow is in the Arctic Circle. The Durbin-Watson and collinearity tests are satisfactory, and the *R* ^2^ (0.399) along with the* t* statistics and *P* values for the coefficient on Δ[CO_2_] do not imply that changes in [CO_2_] at the foot of Mauna Loa have had “most” (Hegerl et al. [17]) to do with temperature changes there since 1960. Instead, the coefficients on the variables in Table 6 indicate that [H_2_O] accounts for more than 90 per cent of temperature change near where C. D. Keeling began his measurements of the atmospheric concentration of CO_2_ back in 1958.^15^

**Figure 2:**
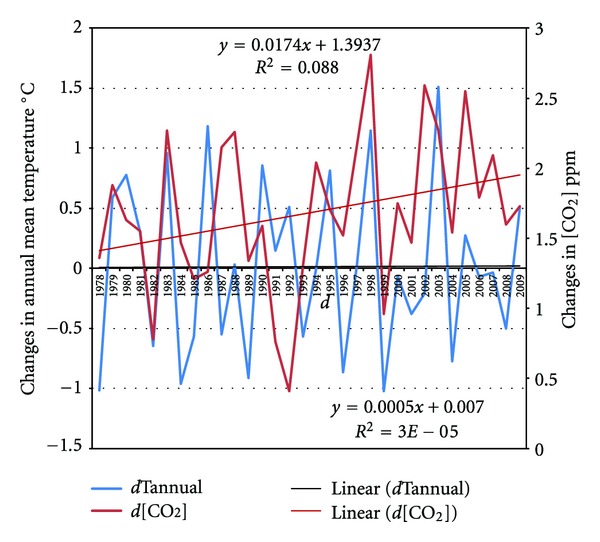
Trends in annual *changes* in annual mean temperatures and average annual [CO_2_] Mauna Loa Slope Observatory 1977–2009. Notes: neither of the trends has good linear fits, with *R* ^2^ < 0.1, and there is in fact no discernible trend in changes in the annual mean temperature at Mauna Loa. Temperature data at Mauna Loa are not included in any of the Hadley-CRU, GCHN, and Gistemp data sets.

I noted above that water vapor is the most potent greenhouse gas because it absorbs strongly in the infra-red region of the light spectrum, first demonstrated by Tyndall [10], despite the conventional view [2] that because the water vapor content of the atmosphere will increase in response to warmer temperatures, water vapor is only a feedback that merely amplifies the climate warming effect due to increased carbon dioxide alone. In reality, the [H_2_O] variable in the NOAA's database proves to be a remarkably powerful determinant of climate variability over the period from 1960 to 2006 not only at Barrow but across all USA, as it is always highly statistically significant at better than the 95% level of confidence for both annual mean minimum and maximum annual temperatures. This is hardly surprising, if only because in reality, as Tans has noted^16^, “global annual evaporation equals ~500,000 billion metric tons. Compare that to fossil CO_2_ emissions of ~8.5 billion ton C/year,” and even the total level of [CO_2_] is only 827 billion tonnes of carbon equivalent. It would seem to be a case of the tail wagging the dog if the additions to [CO_2_] from human burning of hydrocarbon fuels have raised global temperatures enough (just 0.0125°C p.a. since 1950) to generate annual evaporation of 500,000 billion tonnes of [H_2_O], especially when as I have shown here, its role in explaining temperature changes is much less than claimed by the IPCC's Hegerl et al. [17] (see also [5]) (Figure 2). 

## 5. Conclusion

This paper has used basic econometric (multivariate least squares regression) analysis of observational evidence to falsify or confirm two null hypotheses, first that “most” of observed global warming since around 1950 has *not* been “very likely” caused by emissions of noncondensing anthropogenic GHGs [17], and, second, that the noncondensing GHGs do *not* constitute a “control knob” enabling manipulation of global climate. The regression results in the previous Section confirm the first null, as there is no statistically significant evidence to show that increases in anthropogenic GHGs account for any, let alone “most,” of observed global temperature change. 

The second null derives from this statement by Lacis et al. [3].

This assessment comes about as the result of climate modeling experiments which show that it is the noncondensing greenhouse gases such as carbon dioxide, methane, ozone, nitrous oxide, and chlorofluorocarbons that provide the necessary atmospheric temperature structure that ultimately determines the sustainable range for atmospheric water vapor and cloud amounts and thus controls their radiative contribution to the terrestrial greenhouse effect. From this it follows that these noncondensing greenhouse gases provide the temperature environment that is necessary for water vapor and cloud feedback effects to operate, without which the water vapor dominated greenhouse effect would inevitably collapse and plunge the global climate into an icebound Earth state.

Schmidt et al. [2] make a similar claim: “a model simulation performed with zero CO_2_ gives a global mean temperature changes of about −35°C and produces an ice-covered planet (A. Lacis, pers. communication).” These paper's regressions do not invalidate the null that* none* of the Schmidt-Lacis effects is evident when econometric analysis is applied to observations of the most relevant climate variables and instead indicate that the planet's slow warming is mainly associated with the much larger primary rather than feedback changes in atmospheric water vapor, which along with rising [CO_2_] have major social benefits in terms of supporting the rising food production needed to feed a global population now at 7 billion and projected to reach 9 billion by 2050 [6–9]. This may imply the demonization of atmospheric CO_2_ by Hegerl et al. [17] and Schmidt et al. [2], as the alleged primary source of rising temperature could be because of the obvious political difficulty in countries like Australia of blaming increasing rainfall for the observed slow increases in global temperatures evident since 1950. 

The basic physical science underlying the results above is very straight forward, despite the misleading claims in Solomon et al. [1] and Trenberth and Fasullo [34].^17^ These and others distinguish between so-called “long-lived” noncondensing GHGs and the certainly short-lived nature of [H_2_O] arising from evaporation created by solar energy, since it is true that condensation and precipitation generally follow evaporation within at most around ten days. But that does not eliminate nonanthropogenic evaporation, for as Lim and Roderick show [35, page 14], the average daily level of basic [H_2_O] is around 3-4 litres per square meter throughout the year^18^. That is a result of the solar radiative forcing of 342 W/sq. meter [1, page 96]. meter. In contrast the total radiative forcing attributable to noncondensing anthropogenic GHGs is only c. 2.6 W/sq. meter [28]. The annual increase in GMT attributable to up to that level of forcing since 1950 has been only 0.0125°C p.a. But the Clausius-Clapeyron relation which defines the maximum partial pressure of water vapour that can be present in a volume of atmosphere in thermodynamic equilibrium implies that would have only trivial effect on [H_2_O]. The maximum is known as the saturation vapour pressure, *e*_*s*_:^19^

(6) $$\begin{array}{c} e_{s}\left(T\right)=6.1094\exp\left(\frac{17.625T}{T+243.04}\right). \end{array}$$

This formula suggests that the increase in [H_2_O] attributable to rising GMT of 0.0125°C p.a. that could be accommodated in the atmosphere is only 0.047 per cent p.a., not enough to have any measurable effect on GMT, far less than the 2°C to even 3°C and more claimed by Solomon et al. 2007 or Schmidt et al. 2010 for a doubling of [CO_2_] from the preindustrial level of 280 ppm. 

The data underlying my regressions showing that in general variations in [H_2_O] account for as much as 90 per cent of observed changes in temperature both globally and *in situ* suggest that such variations are far larger than those indicated here by Clausius Clapeyron. Thus both my regressions and Clausius Clapeyron fail to invalidate the nulls of the hypotheses advanced by Hegerl et al. [17], Lacis et al. [3], and Schmidt et al. [2]. Consequently it is far from certain that managing the level of atmospheric carbon dioxide concentration really is a meaningful “control knob.” 

## Supplementary Material

This Supplement provides further regression results for data at various locations in support of the results reported in the main text. In addition to the urls of the data sources cited in Table S.4, this author has archived all the data for the locations analyzed here and in the main text, and they are available on request to tcurtin at bigblue.net.au.

## Figures and Tables

**Table:** (a)

Regression statistics	
Multiple *R*	0.814
*R* square	0.662
Adjusted *R* Square	0.651
Standard error	12.384
Observations	31
Durbin Watson	1.749

**Table:** (b) ANOVA

	Df	SS	MS	*F*
Regression	1	8718.49	8718.49	56.85
Residual	29	4447.51	153.36	

Total	30	13166		

**Table:** (c)

	Coefficients	Standard error	*t* stat	*P* value
Intercept	−80.581	16.278	−4.950	0.000
Total radiative Forcings	53.584	7.107	7.540	0.000

**Table:** (a)

Regression statistics	
Multiple *R*	0.183
*R* square	0.033
Adjusted *R* square	−0.001
Standard error	16.467
Observations	30
Durbin Watson	2.760

**Table:** (b) ANOVA

	df	SS	MS	*F*
Regression	1	262.454	262.454	0.968
Residual	28	7592.513	271.161	

Total	29	7854.967		

**Table:** (c)

	Coefficients	Standard error	*t* stat	*P* value
Intercept	−10.073	12.601	−0.799	0.431
dTotalRF	339.775	345.366	0.984	0.334

**Table:** (a)

Regression statistics	
Multiple *R*	0.088
*R* square	0.008
Adjusted *R* square	−0.034
Standard error	0.169
Observations	51

**Table:** (b) ANOVA

	df	SS	MS	*F*
Regression	2	0.011	0.005	0.188
Residual	48	1.372	0.029	

Total	50	1.383		

**Table:** (c)

	Coefficients	Standard error	*t* stat	*P* value
Intercept	−0.030	0.084	−0.360	0.721
RF CO_2_	0.038	0.071	0.544	0.589
Δ[H_2_O]	0.017	0.072	0.230	0.819

Sources: ESRL-NOAA and CDIAC.

**Table:** (a)

Regression statistics	
Multiple *R*	0.660
*R* square	0.435
Adjusted *R* square	0.409
Standard error	1.227
Observations	46

**Table:** (b) ANOVA

	df	SS	MS	*F*
Regression	2	49.913	24.956	16.566
Residual	43	64.779	1.506	

Total	45	114.692		

**Table:** (c)

	Coefficients	Standard error	*t* stat	*P* value
Intercept	0.001	0.473	0.002	0.998
Δ [H_2_O]	17.225	3.076	5.600	0.000
Δ [CO_2_]	0.007	0.311	0.021	0.983

Sources: http://rredc.nrel.gov/solar/old_data/nsrdb/1961-1990/dsf/ and http://rredc.nrel.gov/solar/old_data/nsrdb/1991-2005/ For CO_2_: http://www.esrl.noaa.gov/gmd/ccgg/trends/.

**Table:** (a) Summary output. Dependent variable: year-on-year changes in mean maximum temperatures

Regression statistics	
Multiple *R*	0.593
*R* square	0.351
Adjusted *R* square	0.321
Standard error	1.314
Observations	46

**Table:** (b) ANOVA

	*df*	*SS*	*MS*	*F*
Regression	2	40.162	20.081	11.633
Residual	43	74.228	1.726	

Total	45	114.390		

**Table:** (c)

	Coefficients	Standard error	*t* stat	*P* value
Intercept	−0.075	0.743	−0.101	0.920
ΔH_2_O	15.456	3.207	4.820	0.000
RF abs	0.065	0.648	0.100	0.921

Sources: http://rredc.nrel.gov/solar/old_data/nsrdb/1961-1990/dsf/ and http://rredc.nrel.gov/solar/old_data/nsrdb/1991-2005/ for CO_2_: http://www.esrl.noaa.gov/gmd/ccgg/trends/.

**Table:** (a) Summary output. Dependent variable: year on year changes in mean maximum temperatures

Regression Statistics	
Multiple *R*	0.631
*R* square	0.399
Adjusted *R* square	0.356
Standard error	0.565
Observations	46

**Table:** (b) ANOVA

	df	SS	MS	*F*
Regression	3	8.876	2.959	9.281
Residual	42	13.389	0.319	

Total	45	22.265		

**Table:** (c)

	Coefficients	Standard error	*t* stat	*P* value
Intercept	−0.248	0.219	−1.133	0.264
Δ[CO_2_]	0.195	0.145	1.346	0.186
Δ[H_2_O]	2.564	0.548	4.676	0.000
ΔAVGLO	0.001	0.000	3.721	0.001

Durbin-Watson: 2.834				

**Table 7:** Specimen of NOAA Data Base. Point Barrow 1960–2006 (selected solar and atmospheric variables, data on average windspeed and relative humidity, and so forth are also available). 700260 BARROW W POST-W ROGERS AK -9 N71 19 W156 37 10 1012.

1960	AVGLO	AVDIR	AVDIF	AVETR	AETRN	TOT	OPQ	H_2_O	TAU	MAX_*T *	MIN_*T *	AVG_*T *
January	1	28	1	9	399	4.7	3.4	0.31	0.07	−21.89	−28.5	−25.2
February	259	879	174	692	8541	5.1	3.9	0.29	0.08	−24.36	−30.95	−27.66
March	1568	3422	767	2980	15482	4.5	3.1	0.27	0.09	−22.79	−29.52	−26.17
April	3672	5181	1819	6387	21863	5.1	3.8	0.32	0.11	−15.19	−22.82	−19.01
May	4661	2925	3367	9870	29980	8.1	7.4	0.58	0.12	−4.33	−9.8	−7.05
June	4898	3687	3131	11824	31777	7.9	7.1	1.02	0.14	3.49	−1.26	1.13
July	4456	3878	2627	10926	31671	7.7	6.8	1.38	0.14	7.24	0.89	4.08
August	2624	1576	1962	7760	24588	8.9	8.3	1.26	0.13	5.75	0.75	3.26
September	1338	715	1125	4262	17865	9.2	8.7	0.81	0.11	1.01	−2.76	−0.86
October	478	451	413	1450	11513	8.5	7.7	0.46	0.09	−7.74	−12.89	−10.3
November	25	92	21	110	2665	7	6.1	0.32	0.08	−15.85	−21.59	−18.72
December	0	0	0	0	0	0	0	0.29	0	−20.68	−27.31	−24.01

Source: http://rredc.nrel.gov/solar/old_data/nsrdb/1961-1990/dsf/ and http://rredc.nrel.gov/solar/old_data/nsrdb/. AVGLO/DIR/DIF: Average daily total solar radiation for the GLObal horizontal, DlRect normal, and DlFfuse horizontal elements (Wh/m^2^). SDGLO/DIR/DIF: Standard deviation of daily total global, direct, and diffuse solar radiation (see note (2) below) (Wh/m^2^). AVETR & AETRN: Average dally total global horizontal (AVETR) and direct normal (AETRN) extraterrestrial solar radiation (Wh/m^2^). TOT, OPQ, H2O, TAU: Average TOTal and OPaQue sky cover (tenths), precipitable water (cm), and aerosol optical depth (unitless). MAX_*T*, MIN_*T*, AVG_*T*: Average maximum, minimum, and 24-hour temperatures (°C).

## Appendix

AVGLO A joule is the unit of energy. The Watt is the unit of power and equal to a joule per second, or, equivalently, a joule of energy equals a wattsecond, and a watthour, Wh, equals 3.6 kilojoules of energy. The daily average solar radiation at Barrow in June 1991 (per square meter), 3918 Wh, was 14.1 MJ (mega or million joules)—which lies in the range noted here. The IPCC's [1, page 141] radiative forcing power of 2.6 W (per square meter) is equivalent to 0.225 MJ daily of additional energy in the climate system (per square meter). Thus, we might conclude that, according to the IPCC, atmospheric CO_2_ accumulated since preindustrial times exerts 1.6% of the power of the sun on a summer's day in Barrow. More precisely, AVGLO is the average daily total radiation for the “Global” horizontal component of solar radiation (Wh/m^2^). “Global” solar exposure is the total amount of solar energy falling on a horizontal surface. The daily global solar exposure is the total solar energy for a day. Typical values for daily global solar exposure range from 1 to 35 MJ/m^2^ (megajoules per square meter). The values are usually highest in clear sun conditions during the summer, and lowest during winter or very cloudy days… Irradiance is a measure of the rate of energy received per unit area and has units of Watts per square meter (W/m^2^), where 1 Watt (W) is equal to 1 Joule (J) per second. Radiant exposure is a time integral (or sum) of irradiance. Thus a 1 minute radiant exposure is a measure of the energy received per square meter over a period of 1 minute. Therefore, a 1-minute radiant exposure equals mean irradiance (W/m^2^) × 60(s) and has units of joule(s) per square meter (J/m^2^)” (see *Solar Radiation Definitions*, Australian Bureau of Meteorology, 2009). The NOAA/NREL data are hourly averages in Wh/m^2^, so “AVGLO” of 3918 Wh/m^2^ at Point Barrow in June 1991 is equivalent to 94,032 W/m^2^ per day or 65 W/m^2^ per minute. Thus at Barrow the SSR for the year 1960 amounted to a daily average of 2006 Wh/m^2^, or 17,572,560 Watts/m^2^ over the year. The AVGLO data in Table 7 are net of outgoing albedo (reflection of sunlight), and the NOAA's database therefore also includes the gross “Direct” and “Diffuse” components of incoming solar radiation. The source data includes standard deviations for these variables. Note that the IPCC uses W/m^2^ (not Wh/m^2^) for its measures of the (net) *addition* to solar radiation by “radiative forcing” of greenhouse gases, estimated at 2.6 W/m^2^ (per minute), that is, 156 Wh/m^2^, in 2005 [1, page 141]. That may be compared but never be in Solomon et al. 2007, with the AVGLO (*total* net solar radiation) of 3918 Wh/m^2^ at Barrow in June 1991. However, the former is the IPCC's radiative forcing, a change in the balance of the energy fluxes at top of the atmosphere measured in watts per square meter. The latter is the total energy incident on an average day in say June 1991 on a square meter of Barrow measured in watt hours per square meter, but modified by [H_2_O] and other factors like aerosols and albedo. The IPCC's radiative forcing is gross, that is, without taking into account any of the other factors affecting the level of solar radiation actually reaching the surface at Pt Barrow or anywhere else.

H_2_O It is Precipitable water vapour (cm.) The total atmospheric water vapour contained in a vertical column of unit cross-sectional area is extending between any two specified levels, commonly expressed in terms of the height (cm. in Table 7) to which that water substance would stand if completely condensed and collected in a vessel of the same unit cross-section. See also Solomon et al. [1, pages 271–273], where it is stated “global, local, and regional studies all indicate increases in moisture in the atmosphere near the surface.”

TOT and OPQ Opaque sky cover is the amount of sky completely hidden by clouds or obscuring phenomena, while total sky cover includes this plus the amount of sky covered but not concealed (transparent). Sky cover, for any level aloft, is described as thin if the ratio of transparent to total sky cover at and below that level is one-half or more. Sky cover is reported in tenths, so that 0.0 indicates a clear sky and 1.0 (or 10/10) indicates a completely covered sky (excerpt from *Meteorological Glossary*, AMA, accessed 29 September 2010). *En passant,* we note that the presence of more molecules of CO_2_ in the atmosphere could be expected to *decrease *TOT and OPQ, and this could help to explain why rising [CO_2_] in the local data sets examined here tends to have a *negative*, rather than positive, impact on local temperatures (see Supplementary Material).

Tau—Aerosol Optical Depth (AOD or, in NOAA Data Sets, “Tau”) Aerosol optical depth is a quantitative measure of the extinction of solar radiation by aerosol scattering and absorption between the point of observation and the top of the atmosphere. It is a measure of the integrated columnar aerosol load and the single most important parameter for evaluating direct radiative forcing. The optical depth expresses the quantity of light removed from a beam by scattering or absorption during its path through a medium. If *I*_0_ is the intensity of radiation at the source and *I* is the observed intensity after a given path, then optical depth *τ* is defined by the following equation:

(A.1) $$\begin{array}{c} \frac{I}{I_{0}}=e^{-\tau }. \end{array}$$

(From Wikipedia articles “Aerosol Optical Depth” and “Optical Depth,” accessed 28 September 2010.)

## Endnotes

1. Tol and Vellinga [36] used econometric analysis to separate the enhanced greenhouse effect from the influence of the sun at the top of the atmosphere (TOA), while Tol and de Vos [13] used Bayesian analysis. Neither paper considers the role of atmospheric water vapour. Most common is the fingerprint method (e.g. Hegerl and Allen [18]) which claims to produce a human signal by using Global Circulation Models (GCM). But “the fingerprint approach is only applicable for detection of (dis)similarities between patterns; it seems impossible to use it to derive a probability distribution of the climate sensitivity. We use time series analysis. We do not rely on GCM results—at the expense of using an (overly) simple representation of the climate—and show that this allows to estimate a probability distribution of the climate sensitivity” [13, pages 88-89].
2. The textbook by von Storch and Zwiers *Statistical Analysis in Climate Research* [37] offers an advanced treatment but apart from using climate data for illustrative examples does not itself undertake systematic analysis using its own methods. Those methods are also absent from [17].
3. If only two independent variables are specified, “most” must mean more than 50%; if there are three or more, then “most” means that the preferred variable, in this case [CO_2_], must have greater potency than the sum of the others; for example, 40% is not sufficient if the others sum to 60%. If the criterion for “most” is only that [CO_2_] be the single most potent (and significant) of all the variables, that could be only 1% if all the others *individually* each contribute less than 1% but *jointly* account for 99%. In that case the claims by the IPCC's Solomon et al. that increases in [CO_2_] account for “most” observed global warming are consistent with all states of the world.
4. The basic formula for the radiative forcing attributed to rising [CO_2_] is RF = 5.35(LN(CO_2*t*_/280)), where 280 is the preindustrial level of [CO_2_] in ppm., and CO_2*t*_ is the level in the year in question [29].
5. Stott has 11 citations as a lead author in Hegerl et al. [17].
6. Muller et al. [38] provide data showing very large declines in SO_2_ emissions of the US electric power sector between 1990 and 2008. While coal-fired power's CO_2_ emissions increased by nearly 200 billion tons over that period, SO_2_ emissions by the coal-fired power industry fell from 14.28 million tonnes to 5.5 million tonnes, and similarly for NO_*x*_ (7.1 million tonnes to 2.4). These falls should have enhanced the radiative forcing from rising levels of atmospheric CO_2_, but there is no evidence to support that. Kaufmann et al. [39] attribute the “hiatus” in global warming since 2000 to SO_2_ emissions in China, but their estimate of such global emissions at 65 million tonnes p.a. seems trivial relative to annual emissions of over 30 GtCO_2_.
7. “In the mathematical sciences, a stationary process is a stochastic process whose joint probability distribution does not change when shifted in time or space. As a result, parameters such as the mean and variance, if they exist, also do not change over time or position.” (Wikipedia, October 2010).
8. A stationary series *ab initio* is denoted *I*(0), the first differenced as *I*(1), and the second as *I*(2).
9. The US data are available from http://www.esrl.noaa.gov/gmd/ccgg/iadv/.
10. In the Supplementary Material, I report basic regressions of Gistemp's “global temperatures” as a function of the radiative forcing of the level of [CO_2_].
11. Graphing highly autocorrelated time series data showing rising CO_2_ concentrations and rising temperatures is not enough to “prove” that the data support the theory that the former is responsible for the latter.
12. Point Barrow is also an ideal test of Arrhenius' model [40], since he himself claimed that the temperature effects of doubled [CO_2_] would be significantly higher (6.05°C) at Barrow's latitude (71°N) than at the equator (4.95°C) [40, Table VII] for a doubling of [CO_2_], and 3.52°C for just a 50 percent increase in [CO_2_], compared with 3.15°C at the equator [40, Table VII]. Arrhenius' prediction of a 3.52°C rise in temperature at Point Barrow given a 50 percent increase in [CO_2_] implies that its mean annual temperature would have warmed by more than 3.52°C from the actual minus 12.54°C in 1960 to around minus 9.02°C in 2006, whereas the actual so far is minus 10.2°C, a warming of only 2.52°C. At Hilo near the equator, the predicted “warming” from 1960 to 2006 actually turned out to be a cooling of 0.12°C for the same near 40 percent increase in [CO_2_], but consistent with Arrhenius' prediction that warming would be greater at higher latitudes than lower.
13. If the Durbin-Watson statistic is substantially less than 2, there is an evidence of positive serial correlation, “Durbin-Watson statistic,” *Wikipedia*, accessed 26th October 2010.
14. Kaufmann et al. [15, page 257] also adopt first differencing: “To avoid statistical problems associated with the lack of cointegration, we take the first difference of equation (7)
  (A.2) $$\begin{array}{c} \Delta xt=\rho \Delta xt-1+\Delta \varepsilon t+\theta \Delta \text{temp}t+\text{\_}vt. \end{array}$$
  Specifying the concentration equation in first differences eliminates all stochastic trends and therefore allows us to avoid the effects of carbon uptake by the unknown carbon sink(s) and measurement error on statistical estimates for the effect of temperature on concentrations.”
15. See also Curry at al. [30] and Liu et al. [31].
16. Pers. Comm. See also Tans [41]. For more detailed estimates of global evaporation rates, see Lim and Roderick [35].
17. Trenberth [42] states “Water has a short lifetime in the atmosphere of 9 days on average before it is rained out. Carbon dioxide on the other hand, has a long lifetime, over a century, and therefore plays the most important role in climate change while water vapor provides a positive feedback or amplifying effect; the warmer it gets, the more water vapor the atmosphere can hold by about 4% per degree Fahrenheit”. This claim that atmospheric (CO_2_) has a long lifetime, over a century...,” is at variance with Houghton et al. [24] and Houghton [23], which indicate only about 5 years, because around 20 per cent of atmospheric CO_2_ is continuously recycled between the earth's surface and the atmosphere. Moreover, if atmospheric water vapor arising from solar-based evaporation is “rained out” within 9 days, as claimed by Trenberth and Fasullo [34] that must also be true of [H_2_O] attributable to rising temperature via the Clausius-Clapeyron relation (see below).
18. One millimetre of measured precipitation is the equivalent of one litre of rainfall per metre squared. The estimates in Lim and Roderick [35] of average global evaporation/precipitation of up to 1,187 mm. p.a. in 1970–1999 imply evaporation/precipitation of c.4 mm. per day. Thus, the average daily evaporation implies average [H_2_O] of c. 4 litres per day per metre squared. See also Kelly [43].
19. Wikipedia, “Clausius-Clapeyron”, accessed 30 October 2011, and Pierrehumbert et al. [44, page 145].
