# Supplementary material for: Applying Econometrics to the Carbon Dioxide “Control Knob”
Source: ScientificWorldJournal. 2012 May 3;2012:761473. doi: 10.1100/2012/761473 (PMC3354711; doi:10.1100/2012/761473)
Supplement: Supplementary file 1 — This Supplement provides further regression results for data at various locations in support of the results reported in the main text. In addition to the urls of the data sources cited in Table S.4, this author has archived all the data for the locations analyzed here and in the main text, and they are available on request to tcurtin at bigblue.net.au. [file 761473.f1.docx]

**Appendix 2 Supplementary Information**

**1. Additional Global GMT Results**

One obvious explanation for the poor least squares regression results (Table A2.1) when Temperature anomalies are regressed against the expression for the radiative forcing from rising atmospheric CO_2_, i.e. [CO_2_], is the basically linear rise in the latter relative to the highly annual variations changes in the former, which are often best fit with quadratic or higher level polynomials (Fig.A2.1).

**Fig.A2.1 Trends in temperature and atmospheric CO_2_, Mauna Loa**

Note: The [CO_2_] trend line (R^2^=0.9948) and the annual increments of 1.66 ppm contrast with the Annual Mean Temperature trend line (R^2^=0.0883) and annual increments of 0.0157 ^o^C (0.157 per decade, and 1.57 ^o^C per century).

**Table A2.1**

**Regression of Absolute Values of GMT anomalies (as reported by Gistemp) against CDIAC’s [CO_2_] data (absolute ppm), 1959-2009.**

Notes (Table 2.1): The R^2^ is very high at 0.80, and the t-statistic on [CO_2_] looks strong and statistically significant. However the Durbin-Watson test statistic (1.67) is below the usual benchmark of 2.0, suggesting serial autocorrelation. In addition, since in this case there is only one independent variable, the Condition Index confirms the strong apparent correlation between the dependent and independent variables indicated by the adjusted R^2^ of 0.804. However a bivariate regression analysis like this does not provide evidence for or against the claim that anthropogenic effects account for “most” of observed warming, since no non-anthropogenic variables are present.

**Table A2.2**

**Regression of changes in GMT on changes in [CO_2_] 1959-2009 (constant=0)**

The Durbin-Watson at 2.7 (Table 2.2) rules out spurious correlation, but the R^2^ at 0.066 is derisory, implying that 99 percent of changes in GMT are attributable to other causes than changes in [CO_2_]. The *t*-statistic looks promising but the *p*-value is too high (>0.015). That regression set the constant at zero. Changing this, we have the outcome shown in Table A2.3. The adjusted R2 improves to 0.317, but is well short of the 0.5 required by the IPCC’s “most”, as it indicates that nearly 70 percent of observed global warming cannot be explained by changes in [CO_2_] alone. The regression passes the Durbin-Watson and collinearity tests, and the coefficient on changes in [CO_2_] is statistically significant.

**Table A2.3 Regression of changes in GMT on changes in [CO_2_] (constant ≠ 0).**

The failure of the regressions to account for “most” of global warming should not be surprising in the light of the large disparity in the trends for changes in GMT and [CO_2_] evident in Figs.A2.1 and A2.2 where there is a strong linear upward trend in the changes in [CO_2_] but not for changes in Gistemp’s GMT anomalies.

**Fig.A2.2**

**Trends in *Changes* in Global Mean Temperature and the Atmospheric Concentration of Carbon Dioxide**

Sources: NASA-GISS, Gistemp, and CDIAC

Given the failure of tests of the simple IPCC model adequately to explain either the time series of absolute GMT or annual changes thereof, we could expand the model to include a variable reflecting the known global influence of the El Nino-La Nina phenomenon, as measured by the ENSO or MEI. See Table A2.5 for a regression model of changes in Gistemp’s GMT on changes in [CO2] and the ENSO MEI index, showing the latter plays a very statistically significant role.

With the Durbin-Watson at 2.938, all VIF values at less than 2.5, and all Eigenvalues acceptable (well above 0.0), the results in Table 4.3 show the insignificance of the [CO_2_] variable, and confirm the large importance of the atmospheric water vapour [H_2_O], and total sky cover (“TOT”) variables, while the net surface solar radiation variable “AVGLO” is close to being statistically significant (*t=1.9, P=0.06*) (see Appendix 1 for definitions of these variables).

**Appendix A2.2 Selected *in situ* regression results**

The regression using climatic data for New York City (1960-2006) has results consistent with reported in the main text. Once again the [H2O] variable dominates the [CO2] variable.

**Table 2.2.1**  **Regression of climate data, New York City (JFK) 1960-2006**

| **Change in Annual Mean Maximum Temperatures** |  | **t-statistics** | **p-values** |
| --- | --- | --- | --- |
|  |  |  |  |
| Constant |  | -0.39 | 0.70 |
| Change in [CO_2_] |  | 0.14 | 0.89 |
| Change in [H_2_O]  Change in OPQ |  | 9.64  -1.49 | 0.000  0.14 |
| Standard Error  Adjusted R^2^ | 1.86  0.69 |  |  |
| **Durbin-Watson** | **2.692** |  |  |

Sources: <http://rredc.nrel.gov/solar/old-data/nsrdb/1961-90/dsf/data> and [http://rredc.nrel.gov/solar/old-data/nsrdb/1991-2005/statistics/data. For CO_2_](http://rredc.nrel.gov/solar/old-data/nsrdb/1991-2005/statistics/data.%20For%20CO2): <http://www.esrl.noaa.gov/gmd/ccgg/trends/>

**Table A2.2.2 Multivariate regression of climatic variables, Hilo (Hawaii) 1960-2006**

| **Change in Annual Mean Maximum Temperatures** |  | **t-statistics** | **p-values** |
| --- | --- | --- | --- |
|  |  |  |  |
| Constant |  | -1.13 | 0.264 |
| Change in [CO_2_] |  | 1.35 | 0.186 |
| Change in [H_2_O]  Change in Avglo |  | 4.67  3.72 | 0.000  0.000 |
| Standard Error  Adjusted R^2^ | 0.565  0.356 |  |  |
| **Durbin-Watson** | **2.834** |  |  |

Sources: see Table A2.2.1

**Note:** Independent variables in Table 4: ΔCO_2_ = annual changes in atmospheric carbon dioxide at Mauna Loa Slope Observatory; ΔAvglo =change in annual average daily *in situ* total solar radiation, i.e. sum of direct and diffuse radiation less albedo (Wh/m^2^); ΔH_2_O = annual changes in precipitable water vapour (cm).

Using data from Des Moines (Iowa) the regressions again confirm those above for Barrow, Hilo, New York, see Table A2.2.3.

**Table A2.2.3**

**Some determinants of annual mean maximum temperature, Des Moines, Iowa 1960-2006**

| SUMMARY OUTPUT | |  |  |  |
| --- | --- | --- | --- | --- |
| Dependent variable: mean maximum temperature 1960-2006 | | | |  |
| *Regression Statistics* | |  |  |  |
| Multiple R | 0.524 |  |  |  |
| R Square | 0.274 |  |  |  |
| Adjusted R Square | 0.222 |  |  |  |
| Standard Error | 0.973 |  |  |  |
| Observations | 46 |  |  |  |
|  |  |  |  |  |
| ANOVA |  |  |  |  |
|  | *df* | *SS* | *MS* | *F* |
| Regression | 3 | 15.014 | 5.005 | 5.291 |
| Residual | 42 | 39.729 | 0.946 |  |
| Total | 45 | 54.743 |  |  |
|  |  |  |  |  |
|  | *Coefficients* | *Standard Error* | *t Stat* | *P-value* |
| Intercept | -0.283 | 0.383 | -0.739 | 0.464 |
| ΔAVGLO | 0.004 | 0.001 | 3.544 | 0.001 |
| ΔH2O | 3.961 | 1.756 | 2.257 | 0.029 |
| ΔCO2 | 0.204 | 0.253 | 0.807 | 0.424 |

Sources: Table A2.2.1

To end this section, here are the results of analysis of climate data from the Mauna Loa Slope Observatory (MLSO). In Fig.A2.2.1 above I showed the previously unpublished trends in annual mean temperature and [CO_2_] at Mauna Loa from 1977 to 2009. Clearly there is no good fit for the temperature trend (R^2^= 0.088). It is therefore no surprise that regressing temperature change at MLSO against only its own measurements of changes in [CO_2_] yields no correlation (adj.R^2^=0.095), and the coefficient on changes in [CO_2_] is only just statistically significant (0.46 < 0.05) (see Table 7). But this regression leaves out all non-human variables so lends no support to the conclusions of the IPCC’s Solomon *et al.* (2007).Thus, when solar surface radiation at MLSO itself is included as an independent variable, the regression reported in Table A2.2.3 shows its much greater significance and power on changes in temperature relative to changes in [CO_2_] (on which the coefficient is *negative*).

**Table A2.2.4 The Sun and [CO2] (first differences month-on-month) as determinants of changes in mean temperature: Mauna Loa 1977-2009**

Sources: [CO_2_] – CDIAC; Temperatures: [Thomas.Mefford@noaa.gov](mailto:Thomas.Mefford@noaa.gov)

Note. With only two independent variables, the adjusted R^2^ is unsurprisingly low, at 0.293, but this regression passes the Durbin-Watson and collinearity tests, and the coefficients on both independent variables are significant at better than 95 percent. The *negative* coefficient on the monthly changes in [CO_2_] here is to be expected, as there is the well-known intra-annual variability, whereby in the northern hemisphere’s summer, there is a pronounced drop in the concentration of [CO_2_] because of the uptakes thereof by the biosphere.
